# One‐week test‐retest reliability of an unsupervised, online version of the Digit Symbol Modalities task among older adults

**DOI:** 10.1002/alz.092895

**Published:** 2025-01-09

**Authors:** Andrew Hooyman, Kevin Duff, Sydney Y Schaefer

**Affiliations:** ^1^ Arizona State University, Tempe, AZ USA; ^2^ Oregon Health & Science University, Portland, OR USA; ^3^ NIA‐Layton Aging & Alzheimer's Disease Research Center, Portland, OR USA; ^4^ Neurosessments LLC, Tempe, AZ USA

## Abstract

**Background:**

Measuring individual cognitive ability is critical for the diagnosis of neurodegenerative disease and prognosis of neurorehabilitation. Ideally, individual cognitive ability is achieved through an in‐person neuropsychological assessment by a trained and licensed clinician. However, there are several barriers that may prevent a prospective patient from being assessed (e.g., lack of transportation, reduced access to qualified professionals, etc.). Thus, we developed an online, unsupervised version of the digit symbol modalities task (DSMT), a subtest of the Repeatable Battery for the Assessment of Neuropsychological Status (RBANS) that measures individual processing speed. The online DSMT can be performed both via a computer or touchscreen device. The purpose of this experiment was to examine the one‐week test‐retest reliability of the online DSMT among a group of adults 55 years and older.

**Method:**

Sixty‐eight adults (mean age = 59.6 ± 4.8; female = 14, computer/touchscreen = 68/0) completed the initial and one‐week assessment of the online DSMT. Participants were presented with an instructional video on how to complete the assessment and a limited practice set of the DSMT to become familiarized. Presentation of symbol order was the same across each assessment day and between participants. A one‐way model of intraclass correlation for consistency was used to calculate the intraclass correlation coefficient (ICC).

**Result:**

Results indicated an ICC = .68 (p<.001, 95% CI = [.52; .79]). Furthermore, a dependent t‐test between the initial test (mean = 29.2+/‐10.6) and the one‐week test (mean = 30.8+/‐11.5) was not statistically significant (p = .16), which indicates that performance was relatively stable between time‐points. This result is consistent with prior examinations of ‘good’ test‐retest reliability of the DSMT administered in‐person.

**Conclusion:**

Overall, these results indicate that online, unsupervised DSMT performance is consistent across a one‐week period among adults 55 years and older. Furthermore, the transformation of what is commonly a paper‐and‐pencil test into an online assessment allows for the engineering of digital based performance features that may serve as potential digital biomarkers of neurodegeneration. Future research will validate online DSMT performance with other indicators of neurodegenerative disease.

